# Social causality understanding in relation to irrational attitudes and ambiguity intolerance in schizophrenia

**DOI:** 10.1192/j.eurpsy.2021.1406

**Published:** 2021-08-13

**Authors:** E. Sokolova, K. Andreyuk, A. Ryzhov

**Affiliations:** Faculty Of Psychology, Lomonosov MSU, Moscow, Russian Federation

**Keywords:** schizophrénia, magical thinking, tolerance to ambiguity, mentalization

## Abstract

**Introduction:**

The uncertainty of contemporary social contexts fosters suspiciousness and anaclitic anxieties. In the context of interpersonal relationships this manifests in cognitive distortions and magical thinking, specially in the vulnerable populations.

**Objectives:**

To study the ability of understanding social causality and its relation to magical thinking and ambiguity intolerance in schizophrenia and controls.

**Methods:**

Participants were 40 inpatients with paranoid schizophrenia and 40 controls. Understanding of social causality was measured by corresponding SCORS-S scale for Thematic Apperception Test, Magical thinking was measured by SPQ-74 and intolerance to ambiguity by the New Tolerance-Intolerance to ambiguity questionnaires.

**Results:**

The understanding of social causality was less developed in schizophrenia group (mean values 2.28 and 3.28, p<.001). They manifest omissions of psychological aspects, logical faults and inconsistencies in depicting social relationships. Magical thinking was higher in clinical group (4.32 and 2.33, p<0.001). Two measures were significantly (p<0.05) correlated in both groups. Regression analysis indicates that 37.7% of variance of dependent variable ‘understanding of social causality’ (R2=0,377) was predicted by ‘magical thinking’ (-0,398, p<0,001) and ‘tolerance to ambiguity’ (0,412, p<0,001). The overal level of tolerance of ambiguity was higher in control group (52.2 and 61.0, p<0.002).

**Conclusions:**

Tolerance of ambiguity, being more characteristic for normal population, underlies the understanding of social causality. In contrast, the intolerance to interpersonal ambiguity is related to increment of anxiety, failures in cognitive elaboration of interpersonal relationships and leads to superstition and illogical beliefs. This relationship has a heuristic value for understanding what is happening to vulnerable individuals in the context of current COVID pandemic.

